# Mutant p53 regulates LPA signaling through lysophosphatidic acid phosphatase type 6

**DOI:** 10.1038/s41598-019-41352-5

**Published:** 2019-03-26

**Authors:** Agnieszka Chryplewicz, Samantha M. Tienda, Dominik A. Nahotko, Pamela N. Peters, Ernst Lengyel, Mark A. Eckert

**Affiliations:** Department of Obstetrics and Gynecology/Section of Gynecologic Oncology, The University of Chicago, Chicago, Illinois USA

## Abstract

Emerging evidence has indicated that high-grade serous ovarian cancer (HGSOC) originates in the fallopian tube, where the earliest known genetic lesion is the mutation of *TP53*. In addition to such genetic changes, HGSOC is characterized by altered metabolism, including the production of oncogenic lipids such as lysophosphatidic acid (LPA). To understand the crosstalk between *TP53* mutations and LPA signaling, we utilized primary fallopian tube epithelial cells (FTEC) engineered to overexpress mutant p53. We found that gain-of-function (GOF) p53 mutations downregulated the LPA-degrading enzyme lysophosphatidic acid phosphatase type 6 (ACP6), leading to upregulation of focal adhesion signaling in an LPA-dependent manner. Although highly expressed in normal fallopian tube epithelium, ACP6 expression was significantly reduced in ovarian cancer tumors and early *in situ* lesions. Downregulation of ACP6 in ovarian cancer cells was necessary and sufficient to support HGSOC proliferation, adhesion, migration, and invasion. Using mouse models of metastasis, we established that attenuation of ACP6 expression was associated with increased tumor burden. Conversely, overexpression of ACP6 suppressed invasive behavior. These data identify an involvement of oncogenic p53 mutations in LPA signaling and HGSOC progression through regulation of ACP6 expression.

## Introduction

Ovarian cancer (OvCa) progression is a complex, multi-step process that is characterized by the accumulation of mutations and copy-number alterations (CNA)^1,2^. Large-scale cancer genome sequencing projects have found that *TP53* mutations represent the earliest known genetic lesions^3^ and are present in almost all (96%) high-grade serous ovarian cancers (HGSOC)^4^. A majority of patients are diagnosed at an advanced stage, after tumors have metastasized within the peritoneal cavity, which largely explains the high mortality of the disease^5^. Rather than arising from the ovarian surface epithelium, increasing evidence has supported a fallopian tube origin for HGSOC^6,7^. The earliest known precursor is serous tubal intraepithelial carcinoma (STIC) that is characterized by the presence of p53 mutations, disrupted epithelial architecture, increased proliferation, and DNA damage^7–9^.

The p53 transcription factor is a critical tumor suppressor that regulates DNA repair, apoptosis and growth arrest^10,11^. In normal tissues, p53 is expressed at low levels and activated in response to stress signals, such as DNA damage, metabolic stress, oncogene activation or hypoxia^12^. Mutations in the *TP53* gene often result in the accumulation of stable protein that exhibits a gain-of-function (GOF) phenotype, where mutant p53 loses its function as a tumor suppressor and acquires new oncogenic features independent of wild-type p53 function^11,13^. GOF mutations are commonly present within the DNA-binding domain and either impair the transcriptional activity of wild-type p53 (R273, R248) or perturb p53 conformation (R175, G245, R249)^14^.

In addition to genomic changes, HGSOC are characterized by an altered metabolism, which includes the dysregulation of lipid metabolism^15,16^. Lysophosphatidic acid (LPA) is a bioactive phospholipid with tumor-promoting properties that is present at high levels in the plasma and ascites (15-fold higher) of ovarian cancer patients and indicates an adverse patient prognosis^17,18^. LPA engages with specific G protein-coupled receptors (LPA_1−6_ receptors) to mediate intracellular signaling^19^. LPA is produced by autotaxin (ATX) from lysophosphatidylcholine (LPC) and degraded by lipid phosphate phosphatases through dephosphorylation^20^. In cancer both increased LPA production and reduced degradation may lead to its accumulation, promoting tumorigenesis.

Given that *TP53* mutations are ubiquitous in HGSOC and that elevated LPA levels are tumorigenic and present in the ascites fluid of OvCa patients^3,21^, we studied the interplay between these molecular alterations in fallopian tube epithelial cells. In this study, we report that mutant p53 upregulates LPA signaling through downregulation of the LPA-degrading enzyme lysophosphatidic acid phosphatase type 6 (ACP6), a lipid phosphate phosphatase. ACP6 is localized to the mitochondria where it plays a role in the regulation of phospholipid levels^22–24^. Although ACP6 expression is decreased in esophageal tumors^25^, its potential roles in tumorigenesis and cancer progression have not been functionally investigated. In our study, we find that decreased expression of ACP6 regulates focal adhesion assembly, contributing to increased cell motility and disease progression.

## Results

To model the genetic events that occur during HGSOC development from the fallopian tube, wild-type p53 primary human fallopian tube epithelial cells (FTEC, Supplementary Fig. S1a,b) were separately stably infected with human mutant *TP53* harboring missense mutations in the DNA binding domain: R175H, R249S and R273H^4^ (Supplementary Fig. S1c,d). Mutant p53 FTEC retained expression of fallopian tube epithelial markers (PAX8 and WT1)^26^ and maintained tight junctions *in vitro* as shown by junctional β-catenin staining (Supplementary Fig. S1e,f). Because LPA levels are markedly elevated in the ascites and plasma of ovarian cancer patients and given LPA’s pro-tumorigenic function^17,20,21^, we compared levels of LPA secretion between wild-type and mutant p53 cells. We found that mutant p53 FTEC secreted higher levels of LPA compared to wild-type primary human FTEC, with R273H secreting significantly more LPA than the wild-type cells (Supplementary Fig. S1g).

**Figure 1:**
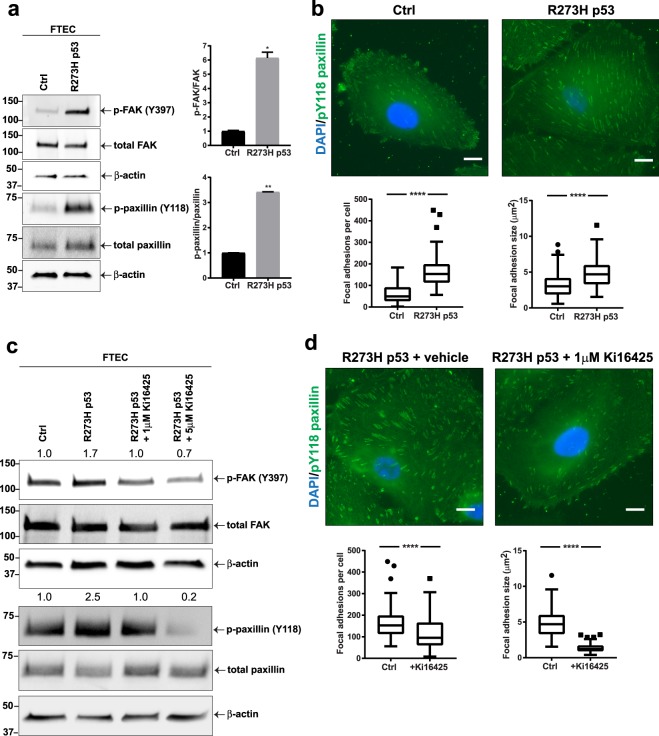
Mutant p53 regulates LPA signaling in fallopian tube epithelial cells (FTEC). (**a**) Immunoblot analysis of tyrosine phosphorylated paxillin (p-paxillin Y118) and FAK (p-FAK Y397) normalized to total protein signal in primary FTEC and FTEC infected with lentiviral p53 R273H (3 independent experiments). (**b**) Analysis of focal adhesions by p-paxillin Y118 immunofluorescence (green) in wild-type (wt) p53 and R273H p53 cells (n = 100 cells). (**c**) Western blot analysis of p-paxillin Y118 and p-FAK Y397 following treatment of R273H p53 mutant FTEC with 1 or 5 µM LPA receptor antagonist (Ki16425) for 1 hr. Quantification of FAK and paxillin phosphorylation (relative to total FAK and paxillin) are presented above the respective immunoblots. (**d**) Immunofluorescence analysis of p-paxillin Y118 to detect focal adhesions in R273H p53 mutant FTEC pretreated with 1 µM LPA receptor inhibitor Ki16425 for 1 hr (n = 100 cells). For (**a**–**d**): *P < 0.05, **P < 0.01, ****P < 0.0001. Error bars are standard error of the mean (SEM).

LPA induces cell migration through induction of focal adhesions and stress fibers^27,28^. In response to LPA, focal adhesion components including FAK and paxillin are tyrosine phosphorylated and activated, which promotes focal adhesion formation and cell motility^29^. We then compared the levels of paxillin (Y118) and FAK (Y397) tyrosine phosphorylation between wild-type and mutant p53 FTEC and found an increase in the R273H mutant p53 cells (Figs 1a and S1h). Expression of R273H p53 increased the number and size of paxillin-containing focal adhesions, as assessed with fluorescence microscopy (Fig. 1b). To determine if LPA signaling was responsible for the phenotype observed in p53 mutant FTEC, we performed a rescue experiment using the established LPA receptor (LPA_1−3_ receptor) antagonist Ki16425^30^. Treatment of mutant p53 FTEC with Ki16425 reduced phosphorylation of paxillin and FAK to the levels observed in wild-type FTEC (Fig. 1c) and reduced the size and number of focal adhesions (Fig. 1d). To investigate the role of LPA receptors in ovarian cancer invasion, we silenced LPA receptors LPA_1_ and LPA_3_ in wild-type and mutant p53 ovarian cancer cell lines with siRNA constructs (Supplementary Fig. S1i). Knockdown of LPA_3_ consistently decreased the levels of phosphorylated paxillin and knockdown of both LPA_1_ and LPA_3_ significantly inhibited *in vitro* invasion, independent of p53 status (Supplementary Fig. S1j,k). Although LPA receptor activity is important for cell motility, LPA receptor expression was not regulated by mutant p53 in FTEC (R273H; Supplementary Fig. S1l). Taken together, these data suggest that R273H p53 mutation induces phosphorylation of paxillin and FAK in an LPA_1−3_ receptor dependent manner, but does not regulate expression of LPA receptors.

**Figure 2:**
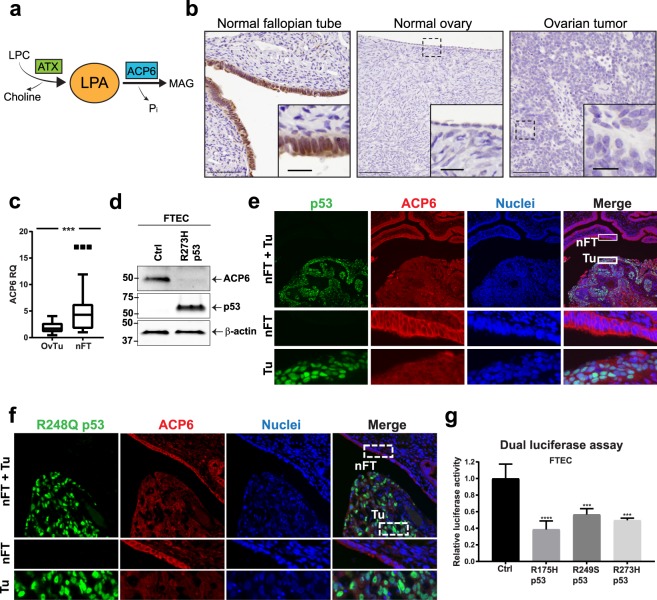
Mutant p53 regulates LPA signaling through ACP6. (**a**) Lysophosphatidic acid (LPA) is produced by autotaxin (ATX) from lysophosphatidylcholine (LPC). Lysophosphatidic acid phosphatase type 6 (ACP6) hydrolyzes LPA to monoacylglycerol (MAG) and inorganic phosphate (P_i_). (**b**) Representative ACP6 immunohistochemistry (IHC) on patient tissues (n = 7, scale bars: 20 and 100 µm) (**c**) qRT-PCR of ACP6 in patient samples: ovarian tumors (OvTu; n = 18 patients) and normal fallopian tube tissues (nFT; n = 12 patients). (**d**) ACP6 protein expression in wild-type (wt) p53 and R273H p53 mutant FTEC. (**e**) Representative immunofluorescence (IF) of ACP6 (red) and p53 (pantropic antibody; green) in tumor and adjacent fallopian tube tissue. (**f**) Immunofluorescence of an HGSOC harboring a R248Q p53 mutation. Analysis of ACP6 (red) and p53 (green). (**g**) ACP6 promoter activity in wild-type (wt) p53 FTEC and R175H, R249S and R273H p53 mutant FTEC as assessed by dual luciferase assay (n = 3, two-way analysis of variance). For (**a**–**g**): ***P < 0.001, ****P < 0.0001. Error bars are SEM.

Given the role of LPA as a strong pro-inflammatory mediator, its homeostasis is tightly regulated through both biosynthesis and degradation (Fig. 2a). We therefore compared the ACP6 expression in benign and malignant ovarian tissues. ACP6 was highly expressed in normal fallopian tube epithelial tissues compared to normal ovaries and ovarian cancer (Figs 2b,c and S2a). To understand if the R273H p53 mutation regulates LPA levels via modifying the expression of ATX, ACP6 or other LPA phosphatases, we examined their transcript levels in wild-type and mutant p53 FTEC. ACP6 RNA and protein expression was significantly reduced in mutant p53 cells, while ATX expression was not affected by R273H p53 (Figs 2d and S2b–d). Other LPA phosphatases, such as LPP1, LPP2 and LPP3, were also not affected by p53 status (Supplementary Fig. 2e). Immunofluorescence staining for ACP6 and p53 found decreased expression of ACP6 in the p53 mutant tumor compartment, while normal fallopian tube epithelial cells demonstrated high expression of ACP6 (Fig. 2e). To investigate if mutant p53 attenuates ACP6 expression in patients with serous OvCa harboring R273H and R248Q mutations, we queried the University of Chicago OvCa tissue bank^31^ to identify patients with HGSOC harboring these specific p53 mutations as assessed by molecular testing. We found significantly decreased expression of ACP6 in p53 mutant tumor compartments of these patients and high ACP6 levels in adjacent normal fallopian tube epithelia (Figs 2f and S2f,g).

As p53 is a transcription factor, we PCR amplified the human ACP6 promoter from normal germline DNA and cloned it into a firefly luciferase reporter vector to directly investigate the regulation of ACP6 transcription. Transient infection of the ACP6 promoter in primary FTEC, fibroblasts, and OvCa cells stably expressing p53 mutants showed that all three p53 mutants (R175H, R249S, R273H) repressed transcription of the ACP6 promoter (Figs 2g and S2h). Stabilization of p53 with gamma irradiation in normal FTEC, HEK293 cells, or HeyA8 cancer cells did not decrease ACP6 levels but rather led to a modest increase in ACP6 expression, indicating that mutant p53 suppressed ACP6 transcription via gain-of-function activities rather than p53 stabilization (Supplementary Fig. S2i–l). Similarly, silencing of p53 expression using small interfering RNA did not alter the expression of ACP6 in both HEK293 cells and transformed HeyA8 cells, confirming that wild-type p53 does not suppress ACP6 expression (Supplementary Fig. S2m–o).

**Figure 3:**
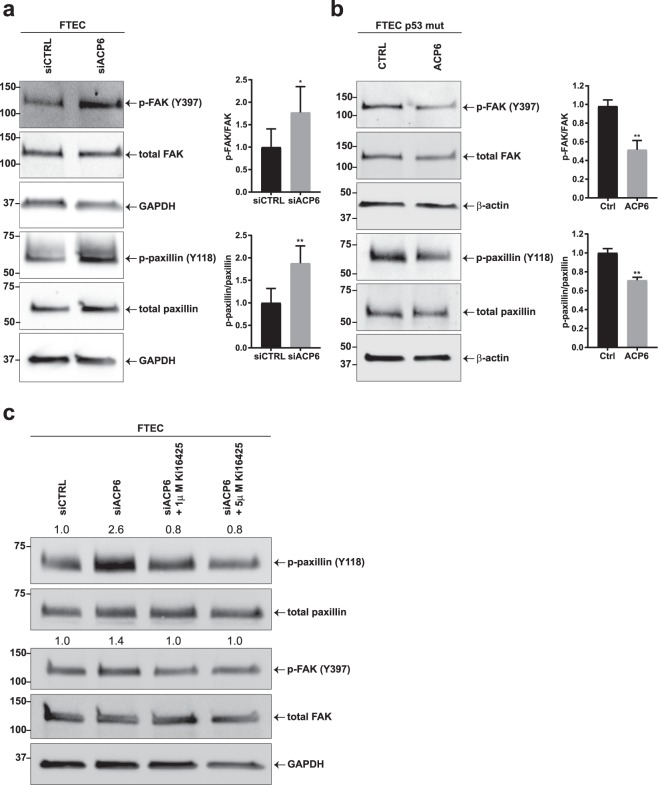
Downregulation of ACP6 regulates LPA signaling. (**a**) Immunoblot analysis and quantification of p-paxillin (Y118) and p-FAK (Y397) normalized to total protein signal in wild-type p53 primary FTEC transfected with an siACP6 or siCTRL construct (n = 3). (**b**) Immunoblot analysis and quantification of focal adhesion proteins in p53 FTEC stably expressing R273H and transiently transfected with ACP6 or a control construct (n = 3). (**c**) Treatment of wild-type (wt) p53 FTEC transiently transfected with siACP6 construct followed by treatment with 2 different concentrations of Ki16425 for 1 hr. Numbers above membranes indicate relative quantification of phosphorylation. For (**a**–**c)**: *P < 0.05, **P < 0.01. Error bars are SEM.

We then sought to determine if attenuation of ACP6 expression by the R273H p53 mutation induces LPA signaling. Transient transfection of FTEC with siACP6 constructs led to increased phosphorylation of focal adhesion proteins (Figs 3a and S3a). Conversely, overexpression of ACP6 in p53 mutant cells led to decreased phosphorylation of FAK and paxillin (Figs 3b and S3b). To determine if the observed effects on focal adhesions were dependent on LPA receptor signaling, we performed a rescue experiment with the LPA receptor antagonist Ki164250: FTEC transiently transfected with siACP6 to mimic the loss of ACP6 in tumor cells that were treated with Ki16425 had reduced paxillin and FAK phosphorylation reversing the effect of ACP6 on focal adhesion signaling (Fig. 3c).

**Figure 4:**
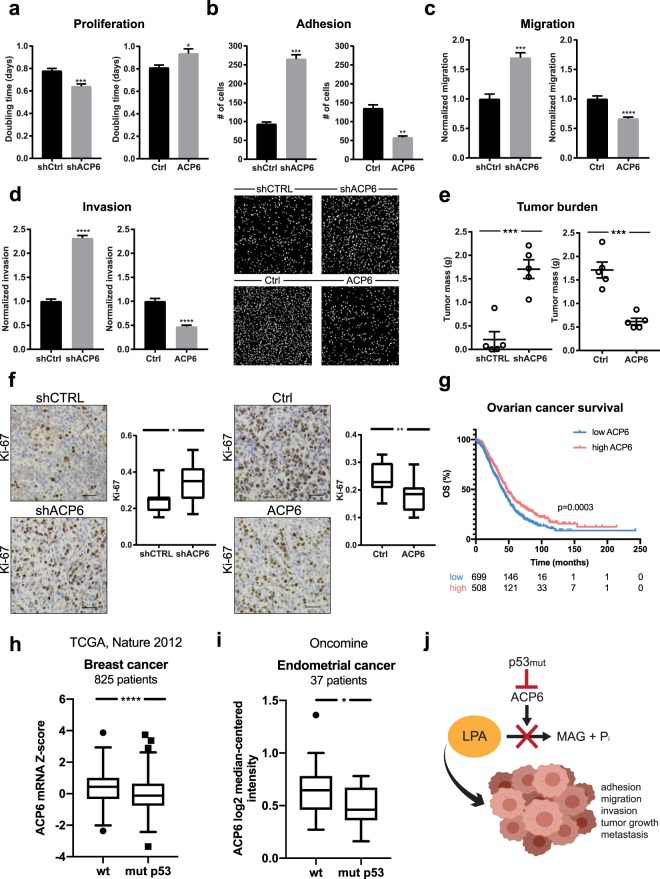
Overexpression of ACP6 suppresses metastasis. Functional assays investigating the effect of ACP6 knockdown or overexpression in HeyA8 cells that were transduced with lentiviral constructs—shACP6, shCTRL, pLX304-ACP6 (ACP6) or empty pLX304 vector (Ctrl)—to downregulate or overexpress ACP6, respectively: (**a**) *in vitro* proliferation (72 hours, n = 10), (**b**) *in vitro* adhesion (30 minutes, n = 3), (**c**) *in vitro* migration (4 hours, n = 4), (**d**) *in vitro* invasion (8 hours, n = 4) and (**e**) *in vivo* tumorigenesis (3 weeks, n = 5). (**f**) Representative Ki-67 immunohistochemistry (IHC) of mouse omental tissue sections (from **e**) and quantification of positive cells (n = 4 slides per group, scale bar: 50 µm). (**g**) Kaplan-Meier overall survival plot for serous ovarian cancer patients finds that low expression of ACP6 is associated with a poor overall survival (40.4 months *versus* 48 months median overall survival for high ACP6 expression). ACP6 expression is significantly reduced in (**h**) breast and (**i**) endometrial cancer patients harboring *TP53* mutations compared to those that maintain wild-type *TP53*. (**j**) Mutant p53 transcriptionally decreases ACP6 expression, leading to LPA accumulation that contributes to a more aggressive ovarian cancer cell phenotype. Monoacylglycerol (MAG). For (**a**–**j)**: *P < 0.05, **P < 0.01, ***P < 0.001, ****P < 0.0001. Error bars are SEM.

To determine if downregulation of ACP6 is necessary and sufficient to promote ovarian cancer progression *in vitro* and *in vivo*, we investigated the effect of ACP6 knockdown and overexpression with the well-characterized^32^ wild-type p53 HeyA8 ovarian cancer cell line (Supplementary Fig. S3c,d). In addition, we studied the phenotypic consequences of ACP6 overexpression in the high-grade serous mutant p53 (R175H) Tyk-nu ovarian cancer cell line (Supplementary Fig. S3e). Downregulation of ACP6 in HeyA8 cells increased cancer cell proliferation, adhesion, migration and invasion. Conversely, overexpression of ACP6 modestly but significantly increased doubling time in HeyA8 cells and markedly inhibited adhesion, migration, and invasion in both Tyk-nu and HeyA8 cell lines (Fig. 4a–d and 4a–c). To understand if ACP6 downregulation is required for tumor growth and metastatic seeding *in vivo*, HeyA8 cells in which ACP6 was either silenced or overexpressed were injected intraperitoneally into nude mice and tumor burden and metastasis evaluated after 3 weeks. This model mimics peritoneal metastasis and avoids confounding effects from variations in primary tumor growth between experimental groups. The same experiment was performed with Tyk-nu cells overexpressing ACP6 or a control construct. Consistent with the *in vitro* functional experiments, the knockdown of ACP6 significantly increased both tumor burden and metastatic spread, while the overexpression of ACP6 reduced tumor burden and metastasis in both HeyA8 and Tyk-nu models (Fig. 4e and 4d–f). ACP6 knockdown or overexpression increased and decreased proliferation, respectively (Ki-67 immunohistochemistry; Fig. 4f).

We next investigated if ACP6 expression predicted patient outcome or tumor grade. Low ACP6 expression was associated with poor overall survival of ovarian cancer patients^33^ (Fig. 4g). We also examined the TCGA^4^ and Oncomine^34^ databases for cancers where the distribution of patients with wild-type and mutant p53 is approximately equal: breast and endometrial cancers. In both cases, ACP6 mRNA expression was significantly downregulated in cancer patients carrying mutations in the *TP53* gene, suggesting that mutant p53 can suppress ACP6 transcription in human tumors (Fig. 4h,i).

## Discussion

Mutation of the *TP53* tumor suppressor is the most common genetic event in epithelial ovarian cancer patients and often leads to a gain of oncogenic functions^4,35^. In addition to genomic alterations, metabolic events are important for ovarian cancer progression with LPA levels being elevated in ascites fluid of patients with advanced ovarian cancer^17,18^. The LPA-rich microenvironment of ovarian cancer can be regulated by either increased LPA production by ATX or decreased LPA degradation by LPA phosphatases^20,36^. LPA signaling then contributes to multiple aspects of cancer dissemination including proliferation, survival, invasion and metastasis^20^.

Herein, we established that mutant p53 downregulates ACP6 expression to increase LPA concentration and signaling, thereby supporting ovarian cancer progression (Fig. 4j). This effect is specific, as other LPA phosphatases^37,38^ with roles in ovarian cancer progression were not regulated by TP53 mutation. Although LPA receptors and ATX play important roles in LPA signaling and homeostasis^39–41^, in our studies expression of these genes was p53-independent. The expression of wild-type p53 did not downregulate ACP6 expression in non-transformed or cancer cells, suggesting that transcriptional repression of ACP6 is due to an oncogenic gain-of-function of p53.

In addition to promoting progression in *in vitro* and *in vivo* model systems, ACP6 expression also correlated with overall survival in serous ovarian cancer patients. Previously, Ando *et al*. found low ACP6 expression to be a marker of poor prognosis in esophageal squamous cell carcinoma (ESCC)^25^. In esophageal cancer, decreased ACP6 levels contribute to tumor growth and lymph node metastasis and ACP6 could have potential tumor suppressive functions. However, the biology of locally invasive ESCC is very different from metastatic HGSOC that disseminates through ascites^5^.

In our study, the most common p53 mutations (R175H, R249S and R273H) all suppressed ACP6 expression. The specific targets of each mutant may be a result of their distinct conformation dependent on the p53 protein domain affected mutation site and nature of the amino acid substitution^42^. These structural changes may influence binding and interactions with other proteins or DNA regulatory regions, and therefore lead to distinct transcriptional repertoires for different p53 mutants^11,43^. All three p53 mutations induced paxillin and FAK phosphorylation despite the fact that R273H mutations most significantly increased LPA concentrations. This may be due to crosstalk between multiple genes differentially expressed in response to each mutant p53 protein. A systematic study of key p53 mutants with regards to gene expression, chromatin architecture, and p53 binding sites in physiologically relevant systems will be essential to deconvolute the disparate functions of p53 mutations.

We find that mutant p53-driven transcriptional downregulation of the LPA phosphatase ACP6 promotes autocrine LPA signaling and focal adhesion signaling. These effects were mediated through phosphorylation of FAK and paxillin, contributing to pro-migratory and pro-invasive phenotypes. FAK is highly upregulated in ovarian cancer patients and is associated with metastasis and poor survival. Indeed, its inhibition has been suggested as a viable therapeutic strategy in advanced HGSOC^44,45^. Our results provide new insight into the crosstalk between GOF p53 mutations and LPA signaling in cancer progression, and indicate that downregulation of ACP6 expression is an early event in HGSOC development, with loss of ACP6 expression observed in *in situ* ovarian cancer. Similar to other studies in ovarian^46,47^, breast^48^ and lung cancer^49^, we find that mutant p53 plays central roles, not only in tumor initiation, but also by priming cancer cells for metastasis. Since mutant p53 mediates interactions between mesothelial and cancer cells during metastasis^46^, ACP6 downregulation followed by increased LPA signaling may also regulate bidirectional communication between cancer cells and other components of the HGSOC microenvironment such as immune cells, cancer-associated fibroblasts, adipocytes, or endothelial cells.

## Materials and Methods

### Plasmids and expression

pLenti6/V5-p53_R175H (#22936), pLenti6/V5-p53_R249S (#22935), pLenti6/V5-p53_R273H (#22943) and pLenti-puro (#39481) plasmids were purchased from Addgene (Cambridge, MA, USA). Short hairpin RNA (Mission shRNA) constructs were purchased from Millipore Sigma (St. Louis, MO, USA). pLX304-ACP6 overexpression construct was obtained from DNasu (HsCD00438184; Tempe, AZ, USA). For lentiviral production, 293 T cells were seeded at 1 × 10^6^ cells per 6 cm dish. After 15 hours, cells were transfected with 1 µg expression vector, 0.9 μg pCMV-dR8.2 packaging vector (#8455, Addgene) and 0.1 μg pCMV-VSV-G expression vector (#8454, Addgene) using Lipofectamine 2000 (Invitrogen, Carlsbad, CA, USA) in OptiMEM Reduced Serum Media. Supernatant containing viral particles was collected after 24, 48 and 72 hours, filtered through a 0.8 µm filter and added to cells for 10 hours before selection with blasticidin (4 µg/ml) or puromycin (1 µg/ml). Small interfering RNA (siRNA) pooled constructs were purchased from Dharmacon (Lafayette, CO, USA). For transient transfections, cells were seeded at 2 × 10^5^ cells per well in a 6-well dish and transfected with 5 nmoles of siRNA using Lipofectamine 2000 in OptiMEM Reduced Serum Media.

### Patient samples and cell lines

All human tissue samples were collected with informed consent under approved University of Chicago IRB protocols and in accordance with the Declaration of Helsinki. HeyA8 (G.B. Mills, Oregon Health & Science University, Portland, OR, USA), HEK293 (ATCC, Manassas, VA) and HEK293T (L. Godley, University of Chicago, Chicago, IL, USA) cells were cultured in DMEM supplemented with 10% FBS, MEM non-essential amino acids, MEM vitamins, penicillin and streptomycin. Tyk-nu cells (K. Sawada, Osaka University School of Medicine, Osaka, Japan) were cultured in MEM supplemented with 10% FBS, MEM non-essential amino acids and MEM vitamins. All cell lines were validated with short tandem repeat marker profiling and tested negative for mycoplasma (IDEXX Bioresearch).

### Primary fallopian tube epithelial cells

Fallopian tubes were collected from patients with benign gynecological conditions during surgery. Fallopian tube epithelial cells (FTEC) were isolated and cultured as previously described^1,50^. FTEC were cultured in DMEM/F12 50/50 (Corning) supplemented with 2% Ultroser G (Pall Corporation, Port Washington, NY, USA), penicillin and streptomycin. For LPA receptor antagonist experiments, FTEC were treated with DMSO as a vehicle control or 1 µM and 5 µM Ki16425 (Cayman Chemical, Ann Arbor, MI, USA) for 1 hour at 37 °C. 18:1 LPA (1-(9Z-octadecenyl)-2-hydroxy-sn-glycero-3-phosphate, Avanti Lipids, Alabaster, AL, USA) was reconstituted in 1% BSA. Cells were treated with 1 µM or 5 µM LPA for 30 minutes at 37 °C.

### Tissue immunohistochemistry and immunofluorescence

Tissue specimens were processed and stained as previously described^1^. Slides were stained with primary antibodies (Phospho-Paxillin (Tyr118), 1:100, Cell Signaling; ACP6, 1:50, Abcam; p53 (Pantropic), 1:100, Millipore Sigma; Ki-67, 1:200, Thermo Fisher Scientific) in 1.25% normal horse serum/PBS or 20% goat serum/TBST overnight at 4 °C. For IHC, slides were visualized using VECTASTAIN Elite ABC HRP kit and DAB Substrate Kit (Vector Laboratories) and counterstained with hematoxylin (Thermo Fisher Scientific). For IF, slides were probed with fluorescent secondary antibodies (1:200, Thermo Fisher Scientific) and Hoechst 33258 (1:200, Thermo Fisher Scientific). Images were acquired with a Nikon Eclipse Ti2 microscope and images processed and quantified with Fiji-ImageJ or NIS-Elements HC.

### Immunofluorescence

8-well chamber slides were coated with fibronectin (5 µg/ml) and FTEC plated at 10,000 cells per well. Cells were fixed with 4% paraformaldehyde, permeabilized with 0.5% Triton X-100 and blocked with 20% goat serum for 1 hour at room temperature (RT). Samples were stained with primary antibodies (Phospho-Paxillin (Tyr118), 1:100, Cell Signaling; PAX8, 1:200, Cell Signaling; WT1, 1:400, Thermo Fisher Scientific; β-catenin, 1:200, BD Biosciences) overnight in 20% goat serum at 4 °C. Slides were probed with fluorescently-labeled secondary antibodies (1:200, Thermo Fisher Scientific) and Hoechst 33258 (1:200, Thermo Fisher Scientific) for 1 hour, washed and mounted with ProLong Gold (Thermo Fisher Scientific). Images were acquired with a Nikon Eclipse Ti2 microscope and images processed with Fiji-ImageJ or NIS-Elements HC. Focal adhesion size was used as one of the assembly descriptors because it precisely predicts cell motility independently of surface coverage and cellular composition^51^.

### RT-qPCR

Total RNA was isolated using RNeasy Mini Kits (Qiagen) according to the manufacturer’s protocol. cDNA synthesis was performed with High-Capacity cDNA Reverse Transcription Kits (Thermo Fisher Scientific). qPCR was carried out with TaqMan probes using TaqMan Fast Advanced Master Mix (Thermo Fisher Scientific) or custom primers (IDT; Skokie, IL, USA; ATX forward primer: 5′-ACTTTTGCCGTTGGAGTCAAT; ATX reverse primer: 5′-GGAGTCTGATAGCACTGTAGGA) using Fast SYBR Green Master Mix (Thermo Fisher Scientific) on a *StepOnePlus* PCR System (Applied Biosystems, Foster City, CA, USA) and analyzed using the 2^−ΔΔCt^ method^52^.

### Immunoblots

Immunoblots were performed as previously described^53^. The membranes were probed with primary antibody (Phospho-Paxillin (Tyr118), 1:1000, Cell Signaling; Paxillin, 1:1000, Cell Signaling; Phospho-FAK (Tyr397), 1:1000, Invitrogen; FAK, 1:1000, Millipore Sigma; ACP6, 1:1000, Abcam; p53 (Pantropic), 1:1000, Millipore Sigma; PAX8, 1:1000, Cell Signaling; GAPDH, 1:1000, Cell Signaling; β-actin, 1:1000, Millipore Sigma) in 2% bovine serum albumin and 0.02% sodium azide in TBST and then incubated with HRP-conjugated secondary antibodies (Thermo Fisher Scientific) at 1:5000 dilution in 5% non-fat dry milk in TBST for 1 hour at RT. Proteins were visualized with Clarity Western ECL Substrate (Bio-Rad) or SuperSignal West Femto Substrate (Thermo Fisher Scientific) using a G:Box XT4 (Syngene).

### Dual luciferase assay

For dual luciferase assays, the pRL-TK Renilla luciferase control reporter vector and pGL3-basic luciferase reporter vector were purchased from Promega (Madison, WI, USA). The pGL3-ACP6 vector was constructed by amplification of a 1,388-bp DNA fragment of the ACP6 promoter from normal germline DNA (forward primer: 5′-TAAGCAGAGCTCATCTGGAAACACAGGCTTG; reverse primer; 5′-TAAGCAGCTAGCAGTCTTCTGCGGGCG) and cloned into the pGL3-basic vector following digestion with SacI and NheI (NEB, Ipswich, MA, USA). Cells were seeded at 2 × 10^5^ cells per well in a 6-well dish and transfected with 1 μg pGL3-ACP6 vector and 100 ng pRL-TK Renilla vector using Lipofectamine 2000 in OptiMEM Reduced Serum Media. All transient transfections were carried out in triplicate with untransfected cells as a background control. Cells were harvested after 24 hours and lysed with Passive Lysis Buffer (Promega). Firefly and Renilla luciferase activities were measured according to the manufacturer’s protocol with a Lumat LB 9507 (Berthold Technologies, Germany) luminometer. Firefly luciferase activity was normalized to Renilla luciferase signal.

### LPA ELISA

Fallopian tube epithelial cells were seeded at 2 × 10^6^ cells per well in a 6-well dish. Conditioned media was collected after 24 hours and filtered through a 0.22 μm filter. LPA concentrations were determined using the LPA Assay Kit II (Echelon, San Jose, CA, USA). Samples used in measurements were not diluted. All data points were collected in duplicate.

### Proliferation

HeyA8 cells were seeded at 500 cells per well in a 96-well plate and allowed to adhere for 24 hours. After 24, 48 and 72 hours, cells were washed once with PBS and fixed with freezer-cold methanol. Cells were treated with RNase A for 30 minutes (100 µg/ml, Invitrogen), nuclei labeled with SYBR Safe (1:5000 dilution, Invitrogen) and wells fluorescently imaged with a SpectraMax i3 plate reader. Images were analyzed with SoftMax Pro software and doubling time extracted using exponential growth equations in GraphPad Prism.

### Adhesion

24-well plates were coated with fibronectin (5 µg/ml) and blocked with DMEM containing 10% FBS. 30,000 cells were plated and allowed to adhere for 30 minutes (HeyA8) or 1 hour (Tyk-nu). Plates were washed once with PBS and fixed with freezer-cold methanol. Cells were treated with RNase A, labeled with SYBR Safe and wells fluorescently imaged with a Nikon Eclipse Ti2^53^. Images were analyzed with Fiji-ImageJ.

### Migration

20,000 cells in OptiMEM Reduced Serum Media were added to the top of the filter membrane of an 8.0 µm PET transwell insert (Thermo Fisher Scientific) with complete media (10% FBS in OptiMEM) in the bottom reservoir. After 4 hours (HeyA8) or 18 hours (Tyk-nu) cells were fixed with 4% PFA/PBS, treated with RNase A and labeled with SYBR Safe. Images were acquired with a Nikon Eclipse Ti2 and analyzed with Fiji-ImageJ.

### Invasion

8.0 µm PET cell culture inserts were coated with collagen type I (100 µg/ml) in molecular grade water and dried overnight at RT. Inserts were rehydrated for 2 hours with warm OptiMEM Reduced Serum Media and cells were added to the top chamber at 20,000 cells per insert. 10% FBS in OptiMEM was added to the lower chamber. Following 8 hours (HeyA8) or 24 hours (Tyk-nu) of invasion, cells were fixed with 4% PFA/PBS, treated with RNase A, labeled with SYBR Safe and imaged with a Nikon Eclipse Ti2. Images were analyzed with Fiji-ImageJ.

### Xenograft models

All animal experiments were performed according to a protocol approved by the University of Chicago Institutional Animal Care and Use Committee. Animals were not randomized, nor excluded. Female nude mice (5 mice per group, 6 weeks old, Harlan) were intraperitoneally injected with 5 × 10^5^ HeyA8 cells expressing shCTRL, shACP6, pLX304 (Ctrl) or pLX304-ACP6 (ACP6) constructs. Tyk-nu cells expressing pLX304 (Ctrl) or pLX304-ACP6 (ACP6) constructs were injected at 5 × 10^6^ cells per mouse. Tumor burden was quantified as total intraperitoneal tumor mass after 21 days, including tumor present in the omentum, peritoneum, parametrial fat pads and mesentery. Mice were sacrificed with isoflurane and cervical dislocation and tumors dissected under blinded conditions.

### Statistical analyses

Statistical analyses were performed using Excel and GraphPad Prism 7.01. Survival analysis of serous ovarian cancer patients included the GSE14764, GSE15622, GSE18520, GSE19829, GSE23554, GSE26193, GSE26712, GSE27651, GSE30161, GSE3149, GSE51373, GSE63885, GSE65986, GSE9891, and TCGA datasets^33^. Unless otherwise stated in figure legends, data are reported as mean ± standard error of means. *P* values were calculated with paired Student’s t-test for immunoblots and unpaired Student’s t-test for other assays. For experiments with more than 2 groups, analysis of variation (ANOVA) with Tukey correction was used. *P* values less than 0.05 were considered significant.

## Supplementary information

Supplementary Fig. S1-18
