# Supplementary material for: Mutant p53 regulates LPA signaling through lysophosphatidic acid phosphatase type 6
Source: Sci Rep. 2019 Mar 26;9:5195. doi: 10.1038/s41598-019-41352-5 (PMC6435808; doi:10.1038/s41598-019-41352-5)
Supplement: Supplementary file 1 — Supplementary Fig. S1-18 [file 41598_2019_41352_MOESM1_ESM.pdf]

Mutant p53 regulates LPA signaling through lysophosphatidic acid phosphatase type 6

Agnieszka Chryplewicz[1], Samantha M. Tienda[1], Dominik A. Nahotko[1], Pamela N. Peters[1], Ernst Lengyel[1*], and Mark A. Eckert[1*]

[1]Department of Obstetrics and Gynecology/Section of Gynecologic Oncology, The University of Chicago, Chicago, Illinois, USA.

*Correspondence: elengyel@uchicago.edu or meckert@bsd.uchicago.edu

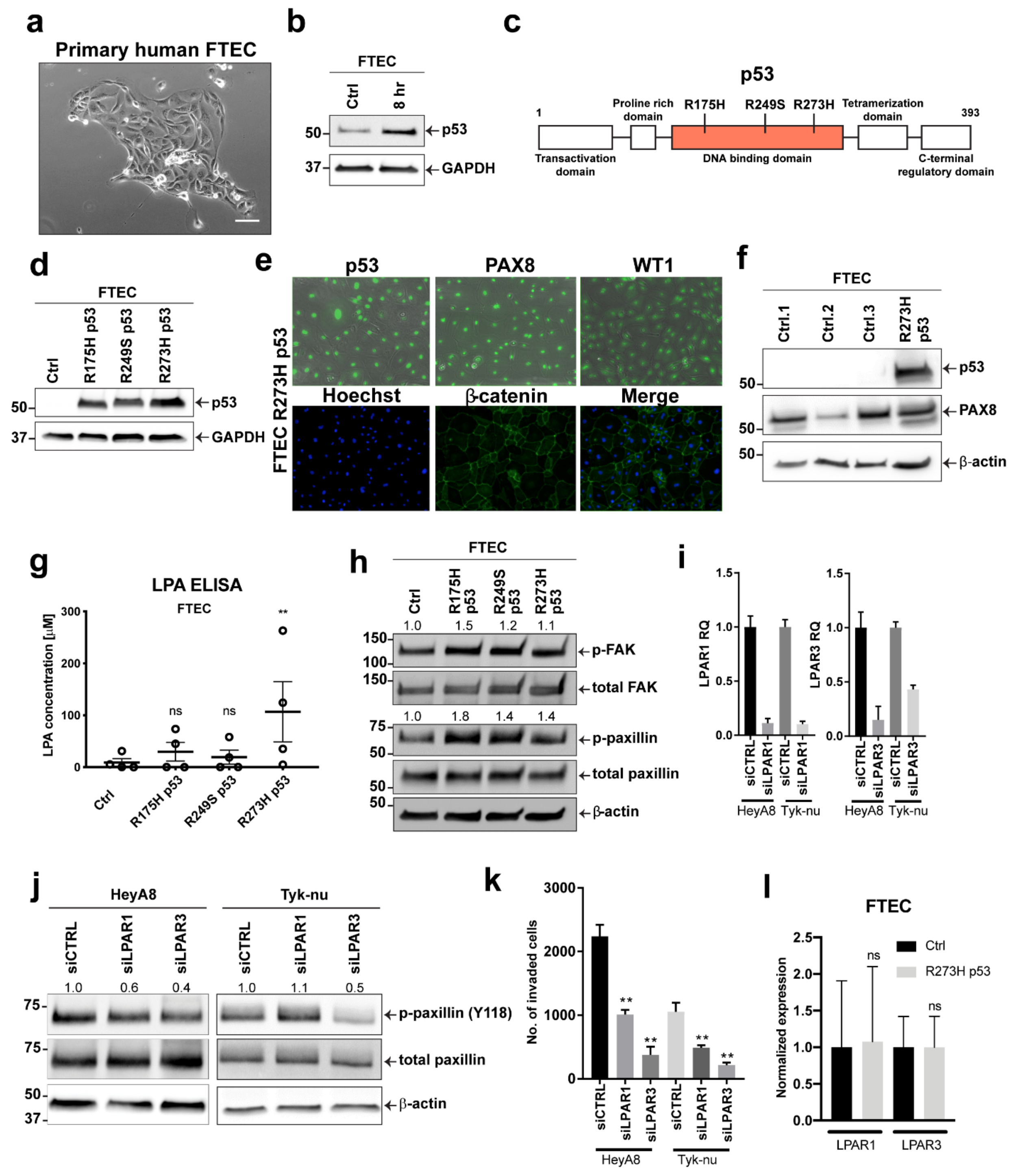

Supplementary Figure S1. Fallopian tube epithelial cells (FTEC) as a model of ovarian cancer evolution.

(**a**) Phase-contrast image of primary human FTEC (scale bar: 50 μm) (**b**) Immunoblot of p53 following irradiation indicates presence of functional wild-type p53 response in primary FTECs. (**c**) Functional domains of p53 protein adapted from Freed-Pastor and Prives *Genes Dev.* 2012. (**d**) Western blot validation of R175H, R249S and R273H p53 mutants. (**e**) Immunofluorescence analysis of fallopian tube epithelial markers (PAX8, WT1) and β-catenin in R273H p53 mutant FTEC. (**f**) Immunoblot for p53 and PAX8 in wild-type (Ctrl.1-3) and mutant p53 FTEC. (**g**) LPA

ELISA: concentration in conditioned culture media of wild-type (wt) and p53 mutant FTEC. Two tailed paired ANOVA (n=4 samples). (**h**) Immunoblot analysis of FAK and paxillin phosphorylation in the indicated gain-of-function p53 FTEC cell clones. (**i**) qPCR validation of LPAR1 and 3 knockdown in indicated cell lines (n=3 replicates). (**j**) Western blot analysis of p-paxillin Y118 in indicated cells. (**k**) *In vitro* invasion of HeyA8 or Tyk-nu cells after knockdown of LPAR1 or 3 (n=3 replicates). (**l**) qPCR analysis of LPAR1 and 3 expression in FTEC expressing R273H mutant p53 (n=3 replicates). For **h** and **j**: Quantification of paxillin or FAK phosphorylation (relative to total paxillin or FAK and normalized to control) are presented above immunoblots.
For **a-l**: **P<0.01, ***P<0.001, ****P<0.0001. Error bars are SEM

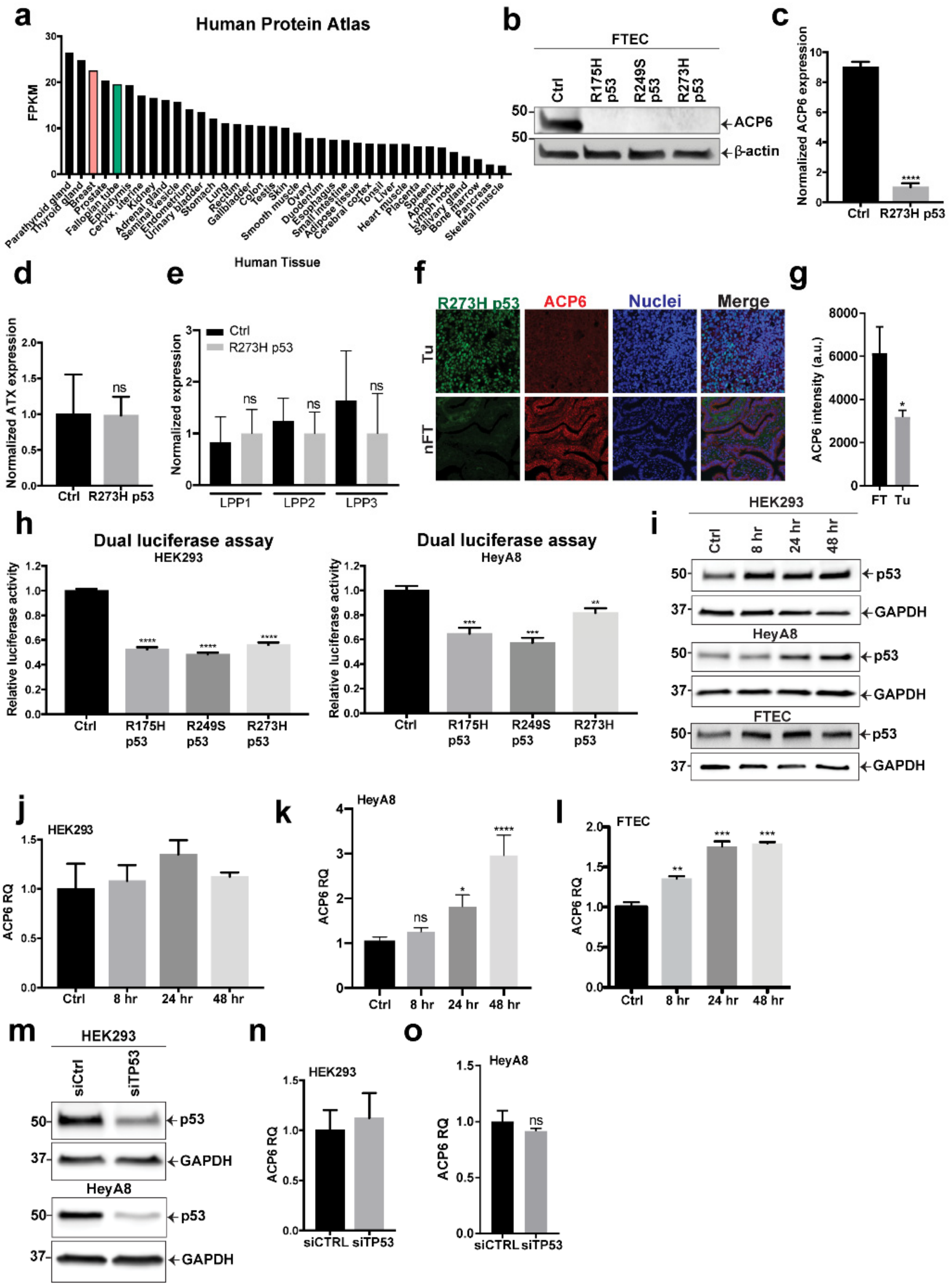

Supplementary Figure S2. Mutant p53 regulates ACP6 expression and activity.

(**a**) Human Protein Atlas analysis of ACP6 mRNA expression across normal tissues. Normal breast tissue in pink and normal fallopian tube in teal. (**b**) Immunoblot analysis of ACP6 expression in R175H, R249S, and R273H p53 FTEC. mRNA expression of (**c**) ACP6, (**d)** ATX, and (**e**) LPP1-3 in control and R273H p53 mutant FTEC (n=3 replicates). (**f**) Immunofluorescence analysis of ACP6 (red) and p53 (pantropic; green) in tumor and normal fallopian tube tissues of a patient with the R273H p53 mutation. (**g**) Quantification of ACP6 staining intensity in normal fallopian tube and tumor tissue from three HGSOC patients. (**h**) ACP6 promoter activity evaluated using dual luciferase assay in wild-type (wt) p53 and R175H, R249S and R273H p53 HeyA8 and HEK293 cells (n=3 independent transfections, two-way analysis of variance). (**i**) Western blot validation of p53 induction by gamma irradiation (10 Gy) after indicated time points in HEK293, HeyA8 and FTEC cells. (**j-l**) qRT-PCR quantification of ACP6 mRNA expression in HEK293, HeyA8 and FTEC cells at indicated time points (n=3 replicates). (**m**) Western blot validation of siTP53 knockdown in HEK293 and HeyA8 cells. (**n-o**) qRT-PCR analysis of ACP6 after siRNA knockdown of ACP6 in HEK293 and HeyA8 cells.
For **a-p**: **P<0.01, ***P<0.001, ****P<0.0001. Error bars are SEM.

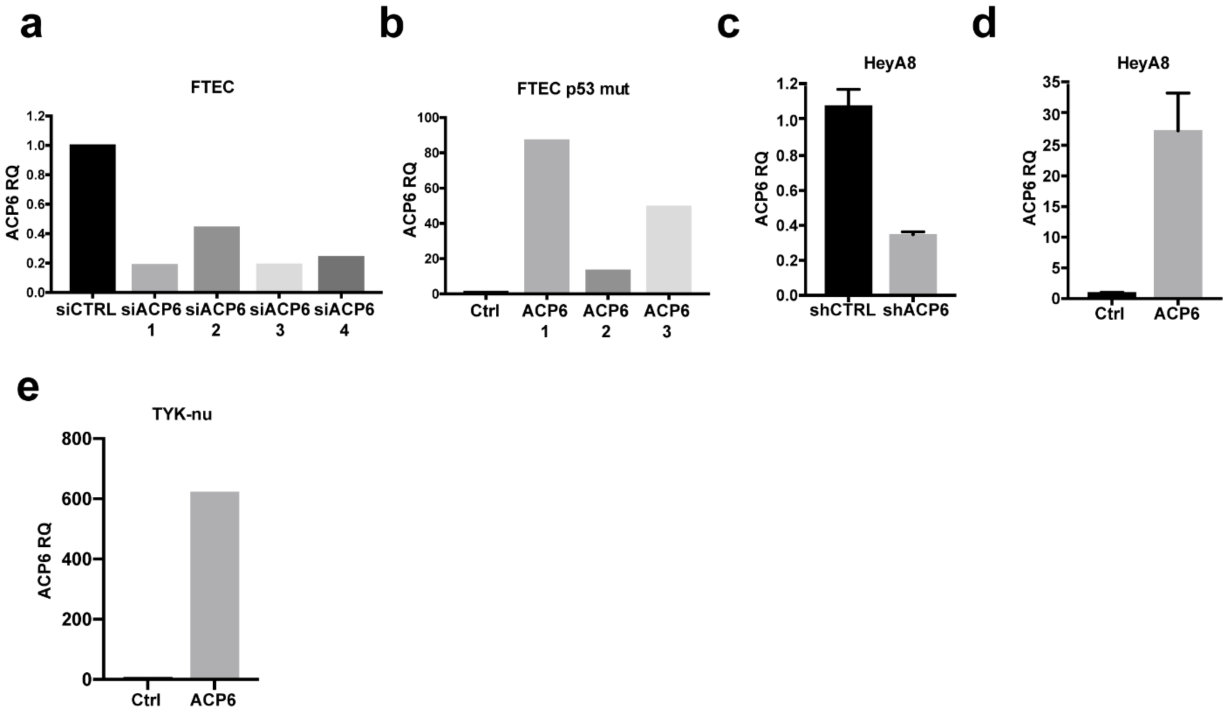

Supplementary Figure S3. Validation of ACP6 expression.
qRT-PCR validation: (**a**) transient ACP6 knockdown with siRNA in primary human FTEC (n=4 patients: siACP6 1-4), (**b**) transient ACP6 overexpression in mutant p53 FTEC (n=3 patients: ACP 1-3), (**c**) stable shACP6 knockdown with shRNA in HeyA8 cells, (**d**) stable ACP6 overexpression in HeyA8 cells, and (**e**) stable ACP6 overexpression in Tyk-nu cells.
Error bars are SEM.

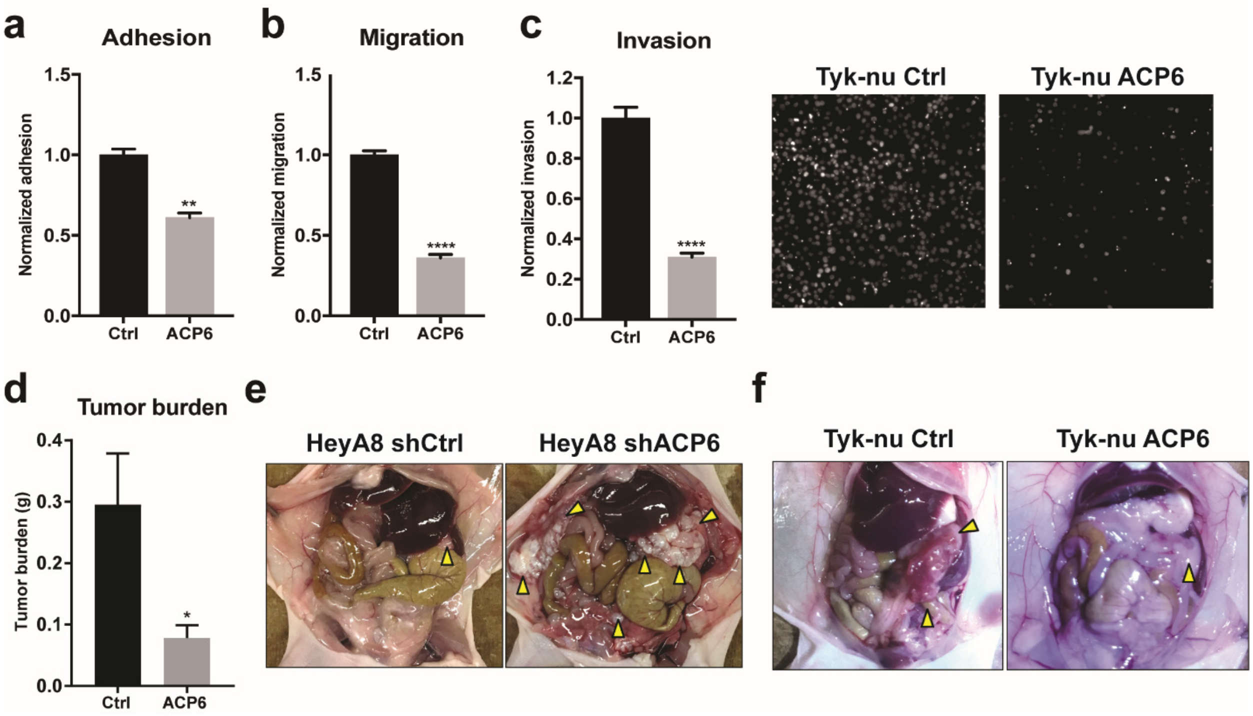

Supplementary Figure S4. ACP6 overexpression inhibits ovarian cancer metastasis. Functional assays investigating the effect of ACP6 overexpression in Tyk-nu cells that were transduced with control or ACP6 overexpression lentiviral constructs: (**a**) *in vitro* adhesion (1 hour, n=3), (**b)** *in vitro* migration (18 hours, n=3) **(c**) *in vitro* invasion (24 hours, n=3) and (**c**) *in vivo* tumorigenesis (3 weeks, n=5 mice per group). Representative images of tumor distribution of (**e**) HeyA8 or (**f**) Tyk-nu cells expressing the indicated ACP6 knockdown or overexpression constructs.

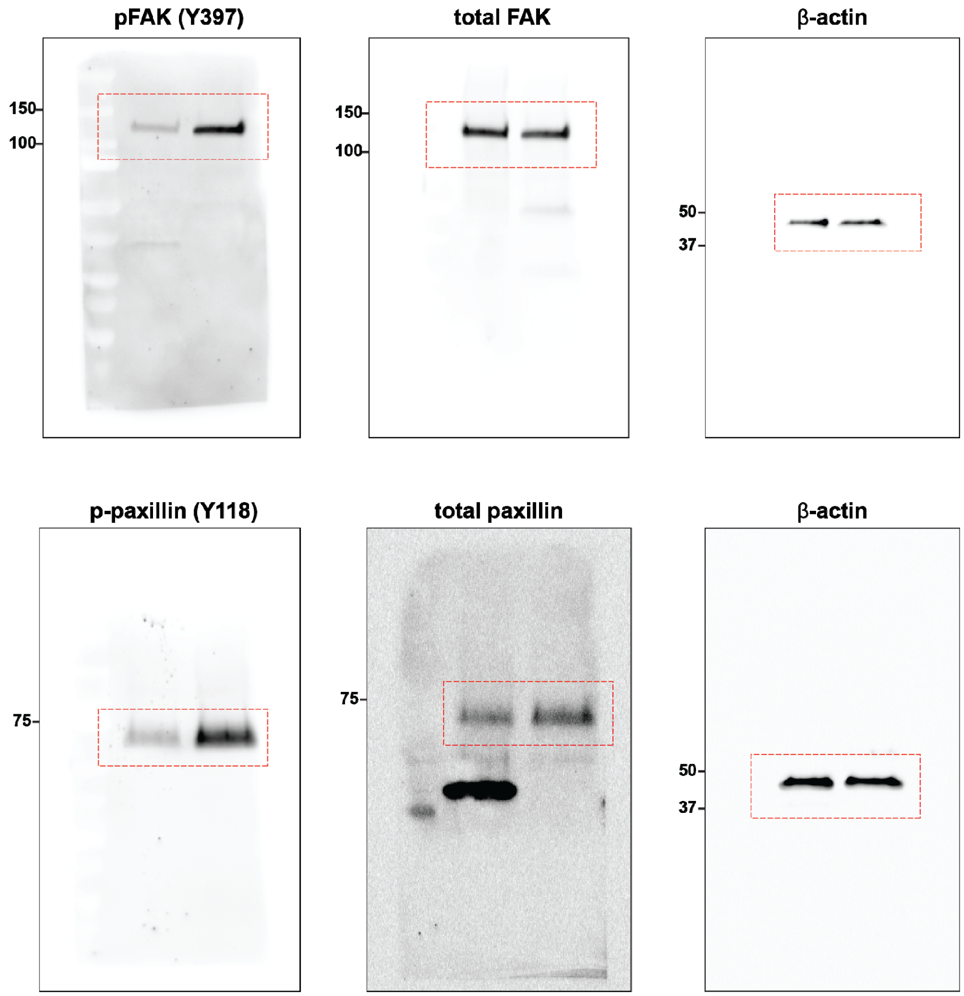

Supplementary Figure S5. Uncropped western blots used in Fig. 1a.

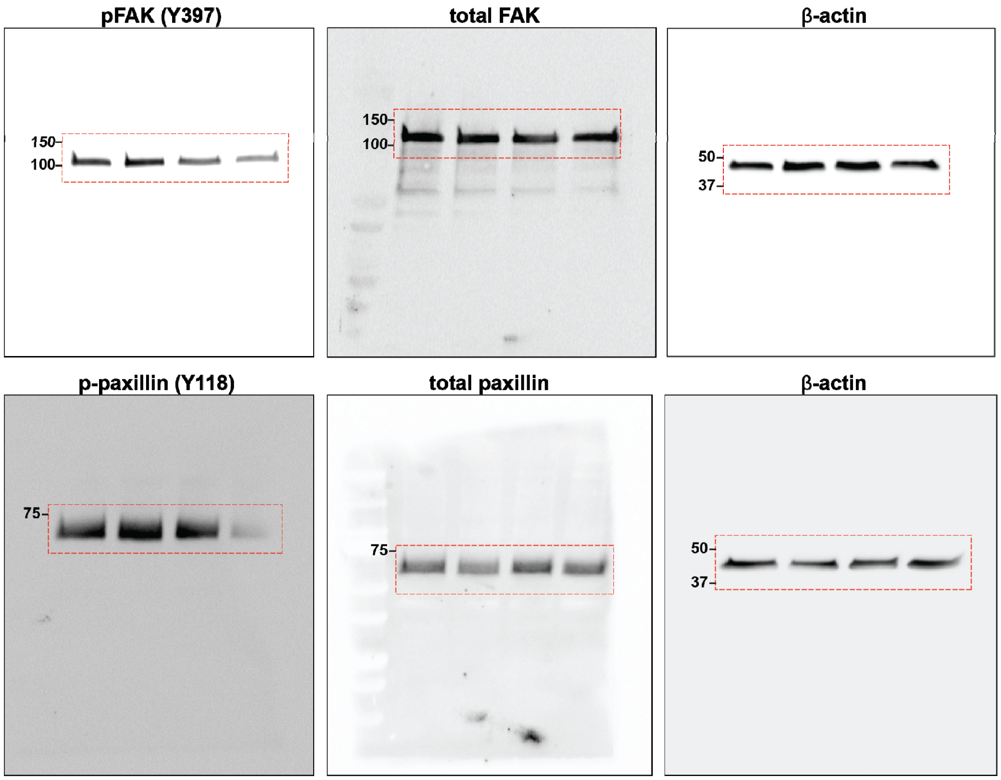

Supplementary Figure S6. Uncropped western blots used in Fig. 1c.

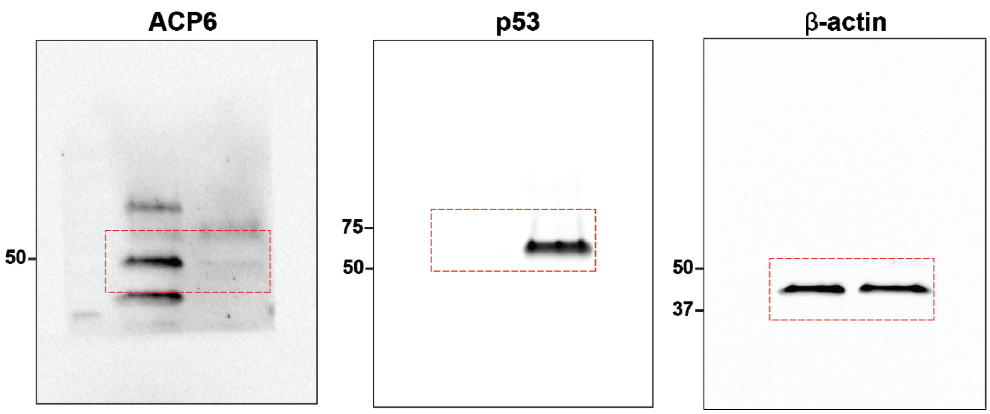

Supplementary Figure S7. Uncropped western blots used in Fig. 2d

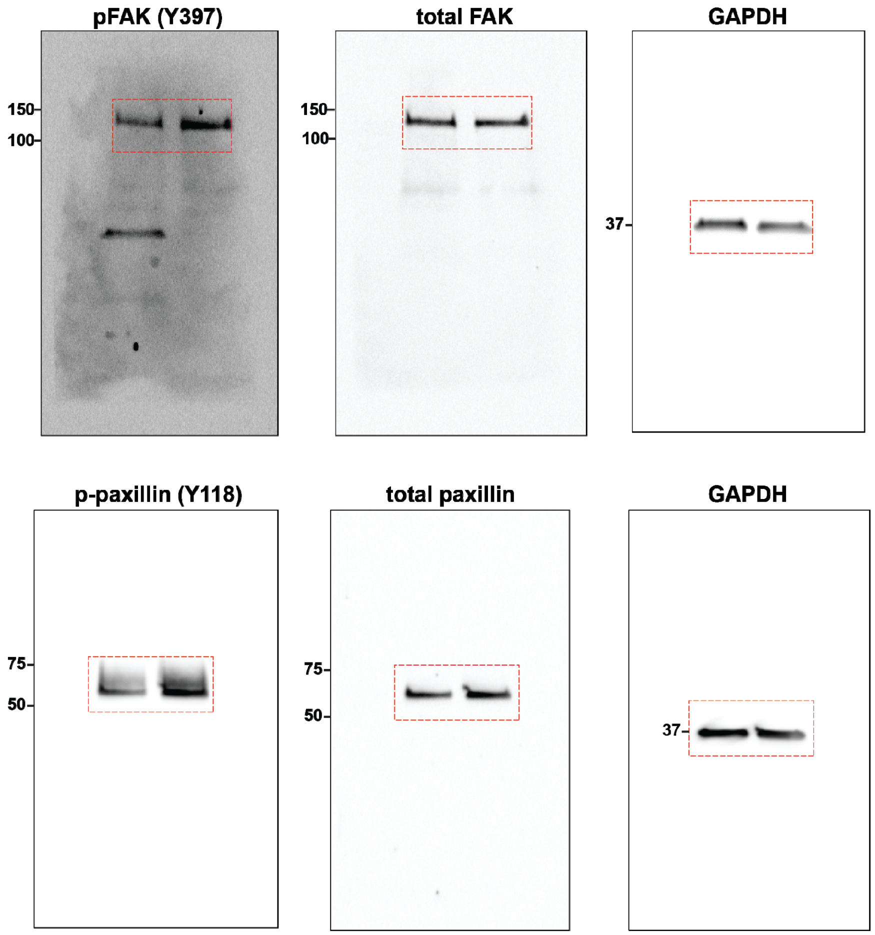

Supplementary Figure S8. Uncropped western blots used in Fig. 3a.

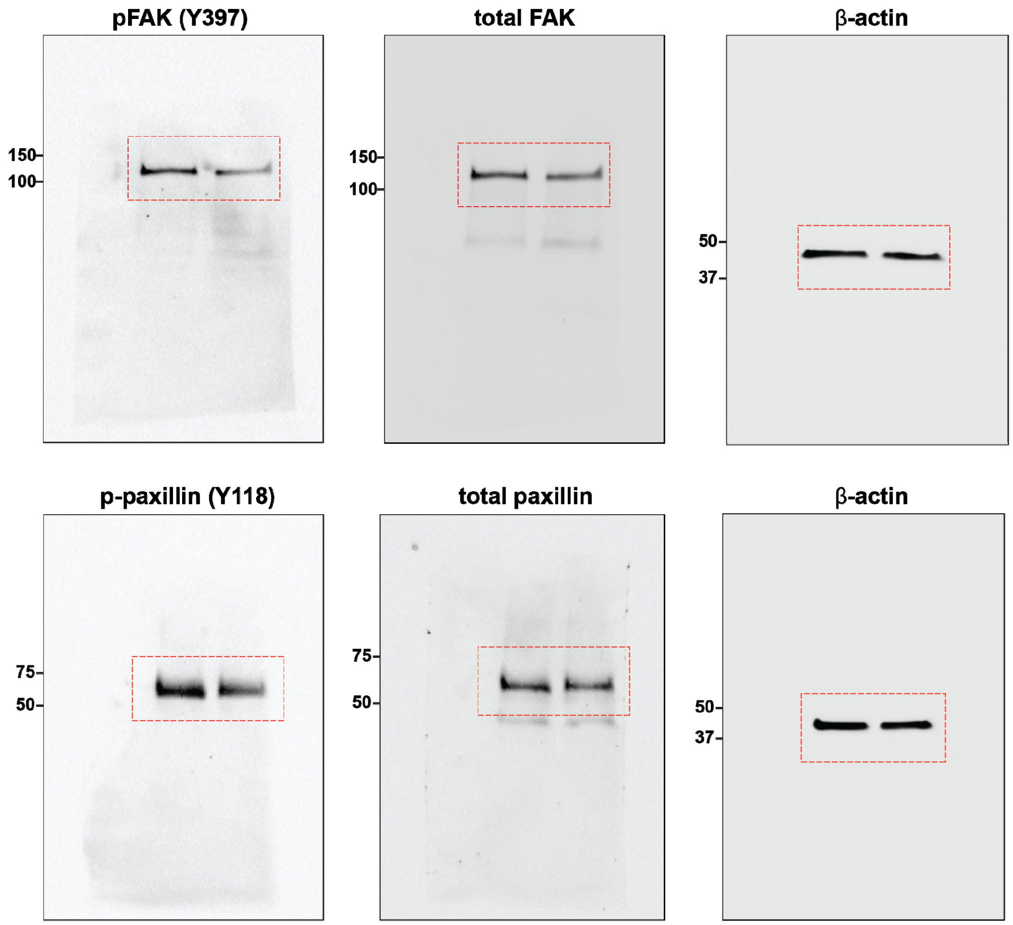

Supplementary Figure S9. Uncropped western blots used in Fig. 3b.

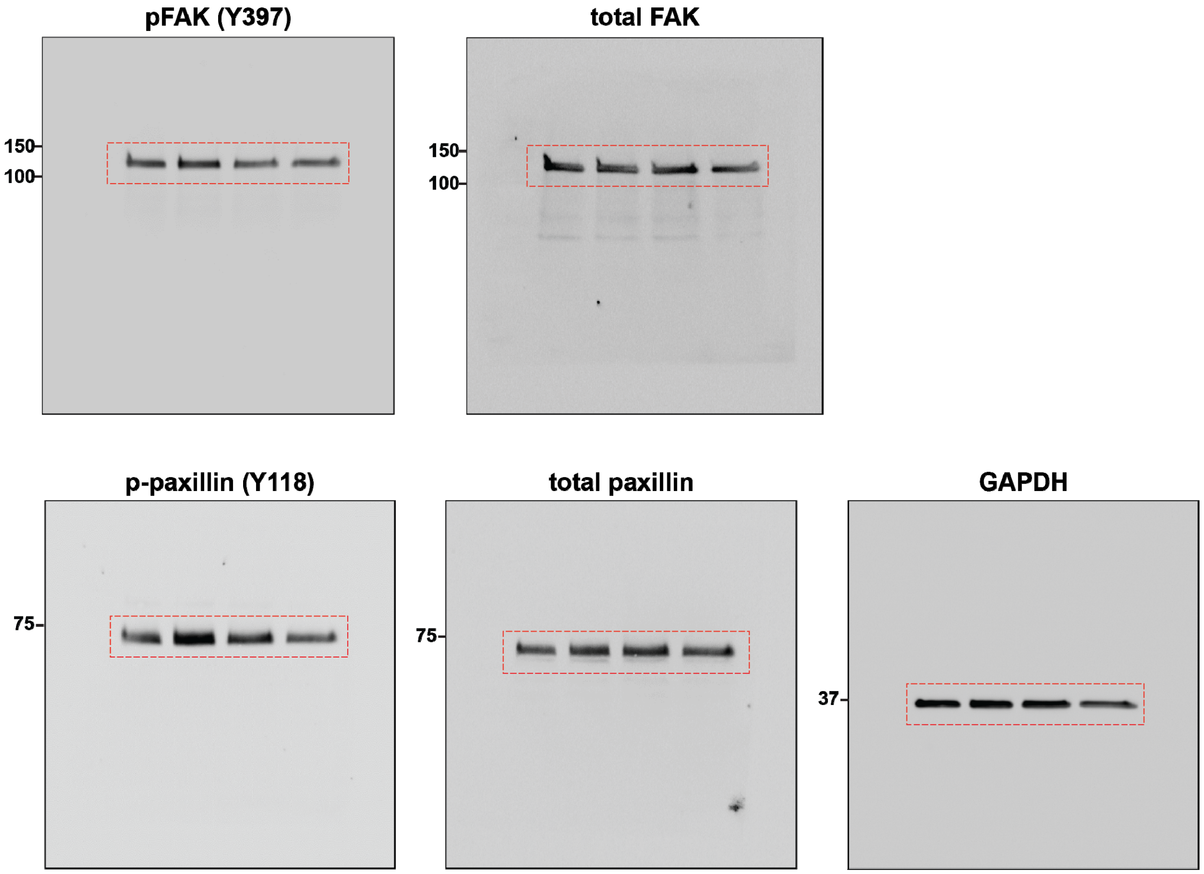

Supplementary Figure S10. Uncropped western blots used in Fig. 3c.

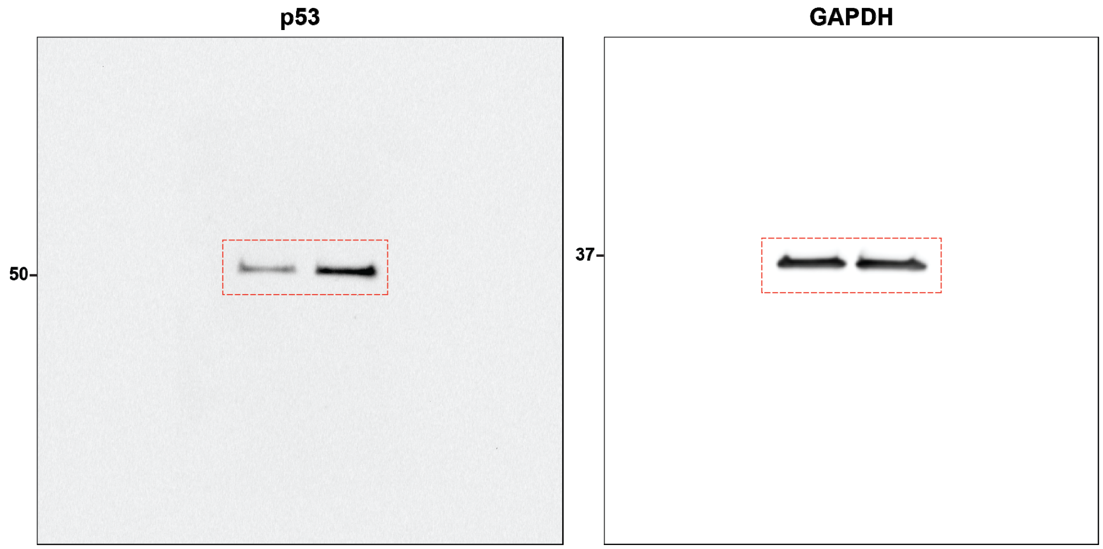

Supplementary Figure S11. Uncropped western blots used in Fig. S1b.

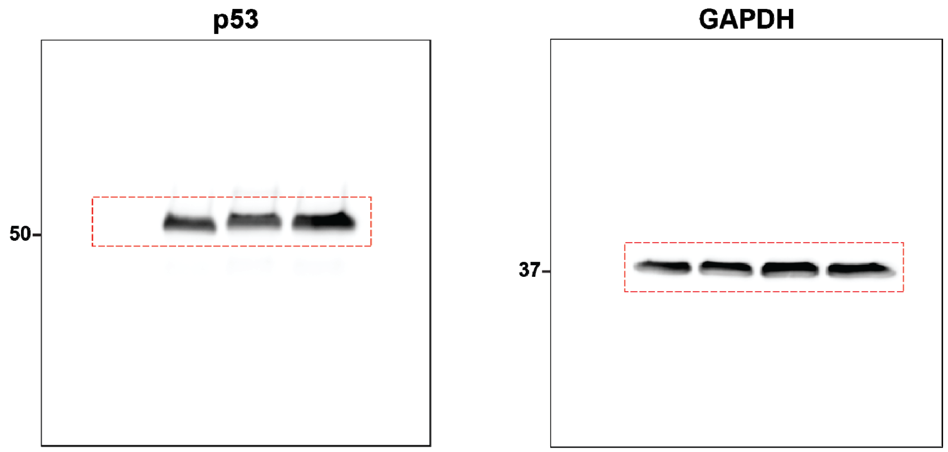

Supplementary Figure S12. Uncropped western blots used in Fig. S1d.

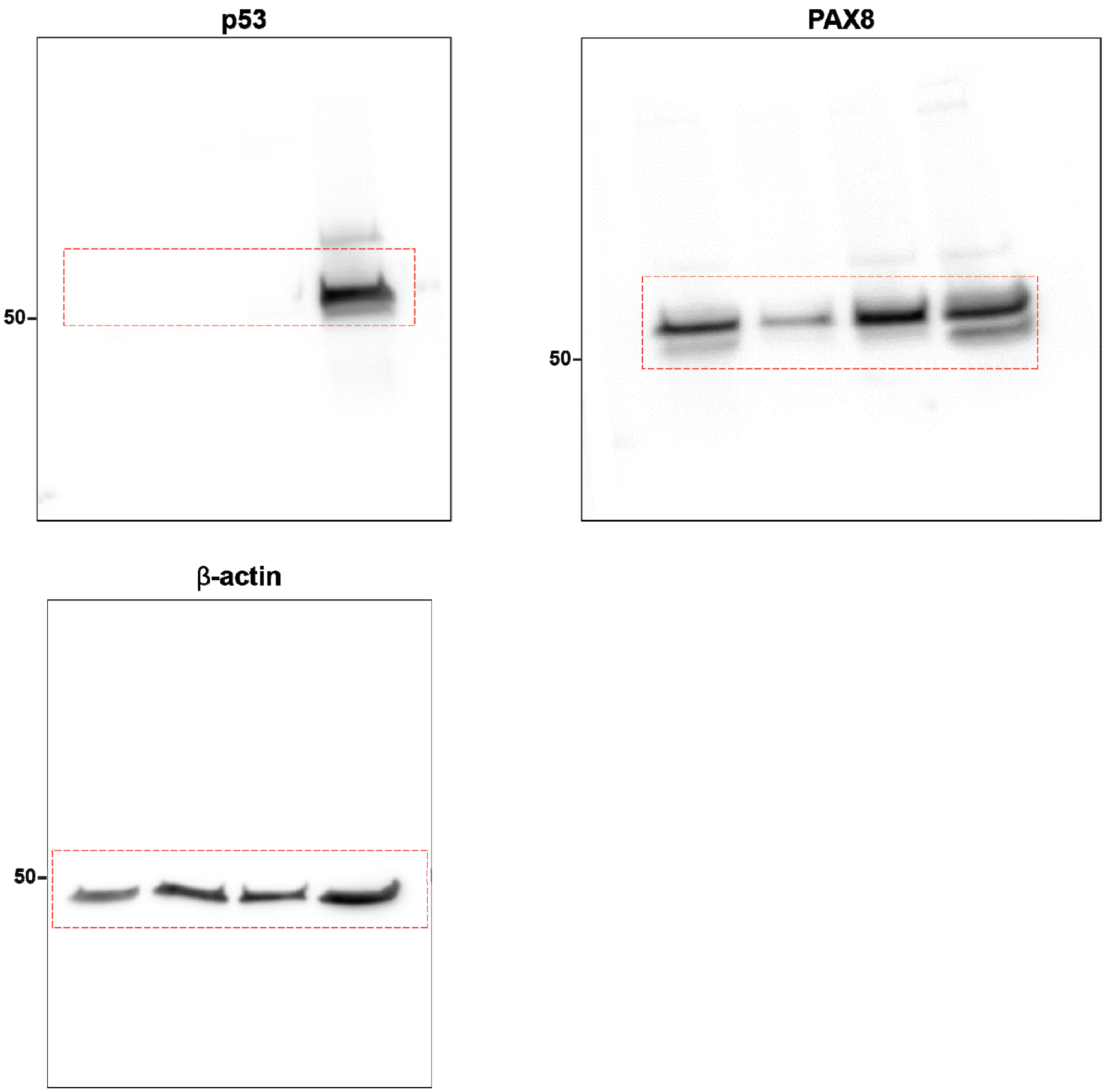

Supplementary Figure S13. Uncropped western blots used in Fig. S1f.

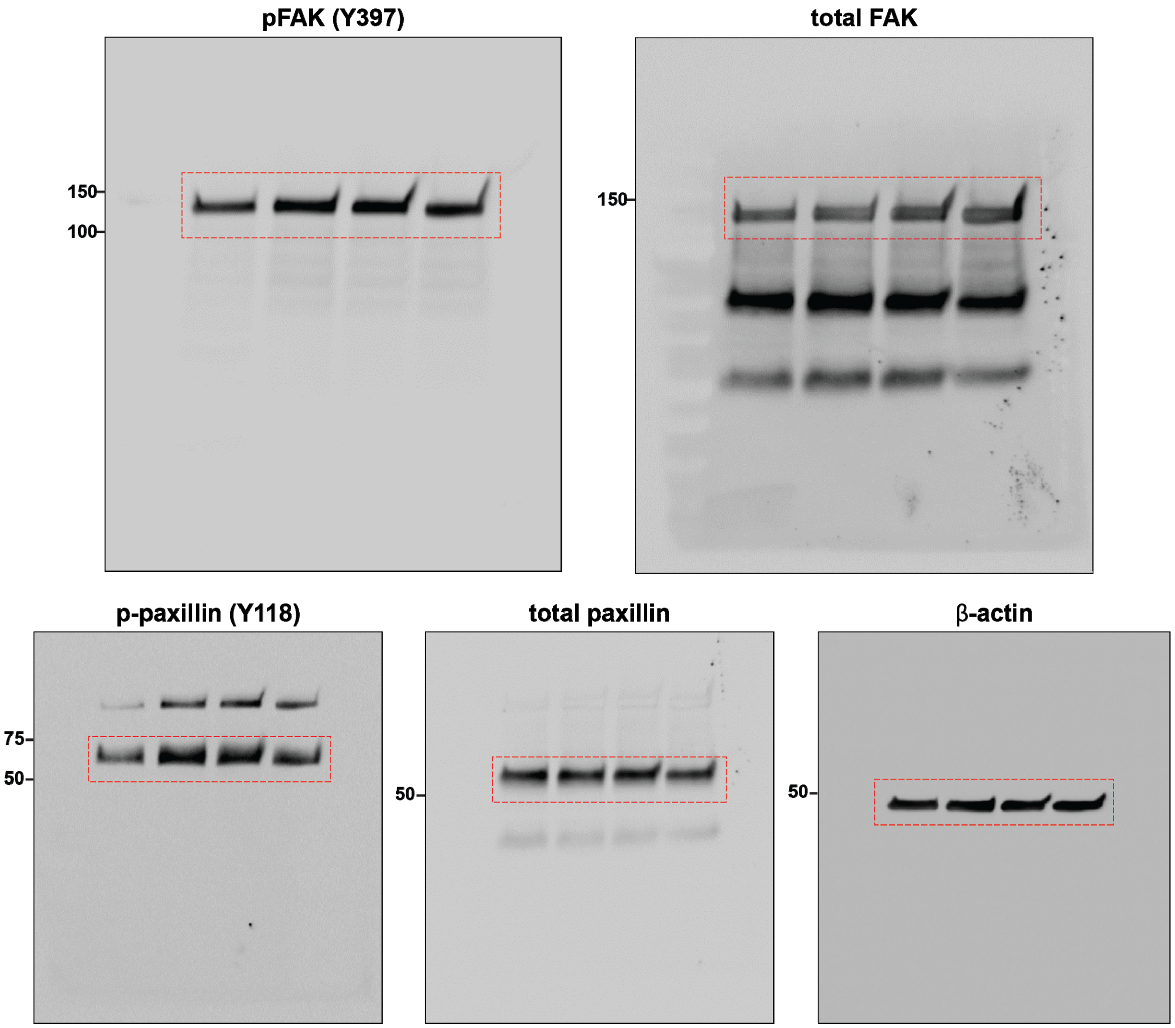

Supplementary Figure S14. Uncropped western blots used in Fig. S1h.

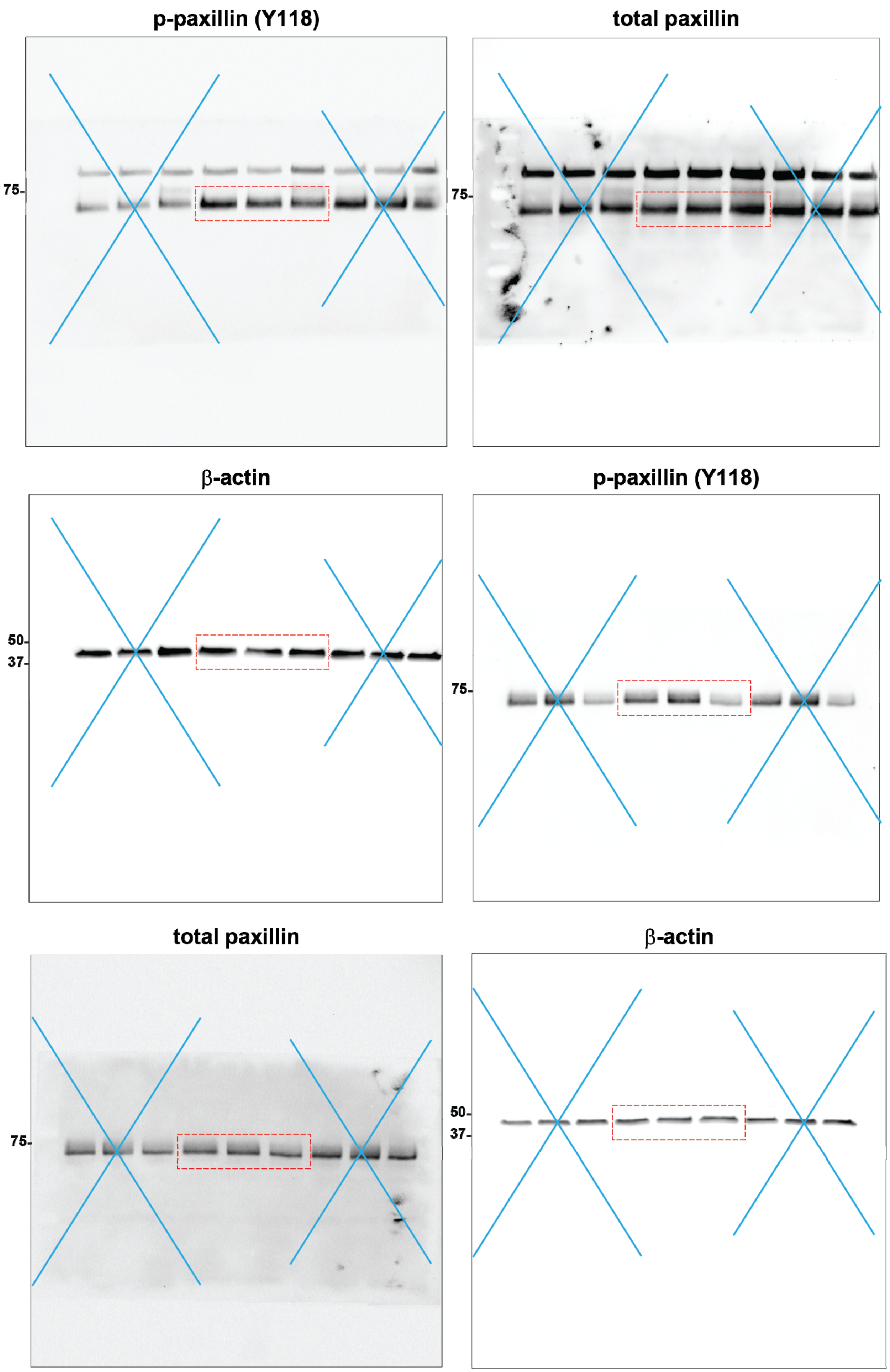

Supplementary Figure S15. Uncropped western blots used in Fig. S1j.

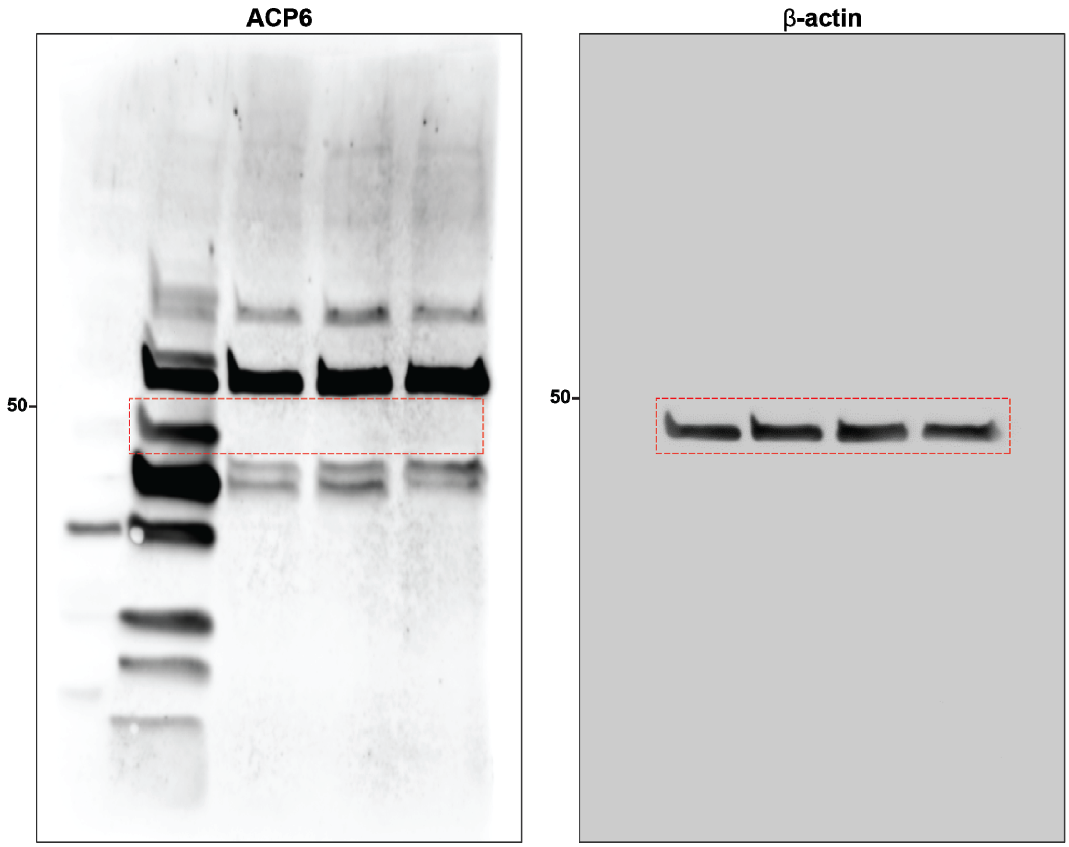

Supplementary Figure S16. Uncropped western blots used in Fig. S2b.

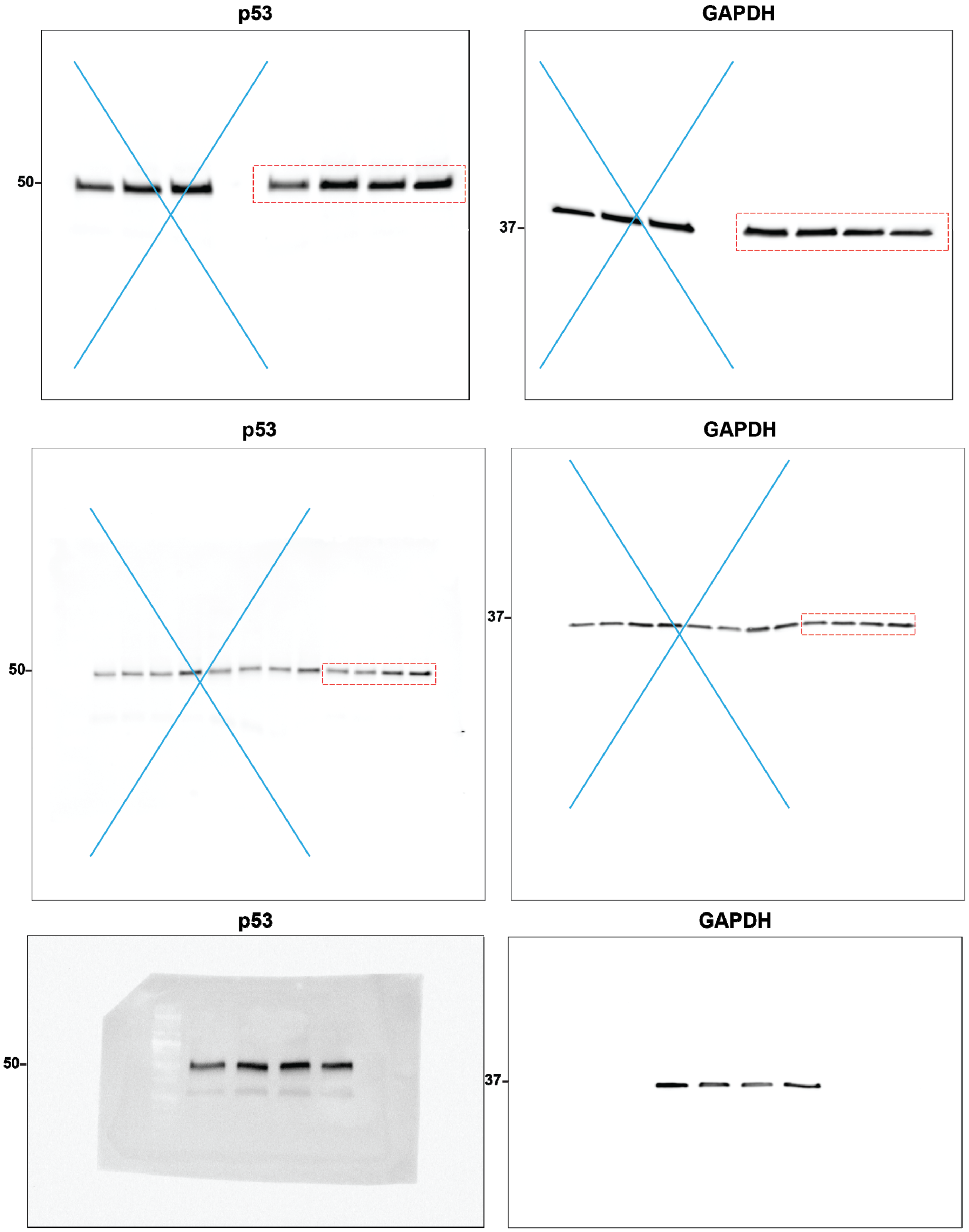

Supplementary Figure S17. Uncropped western blots used in Fig. S2i.

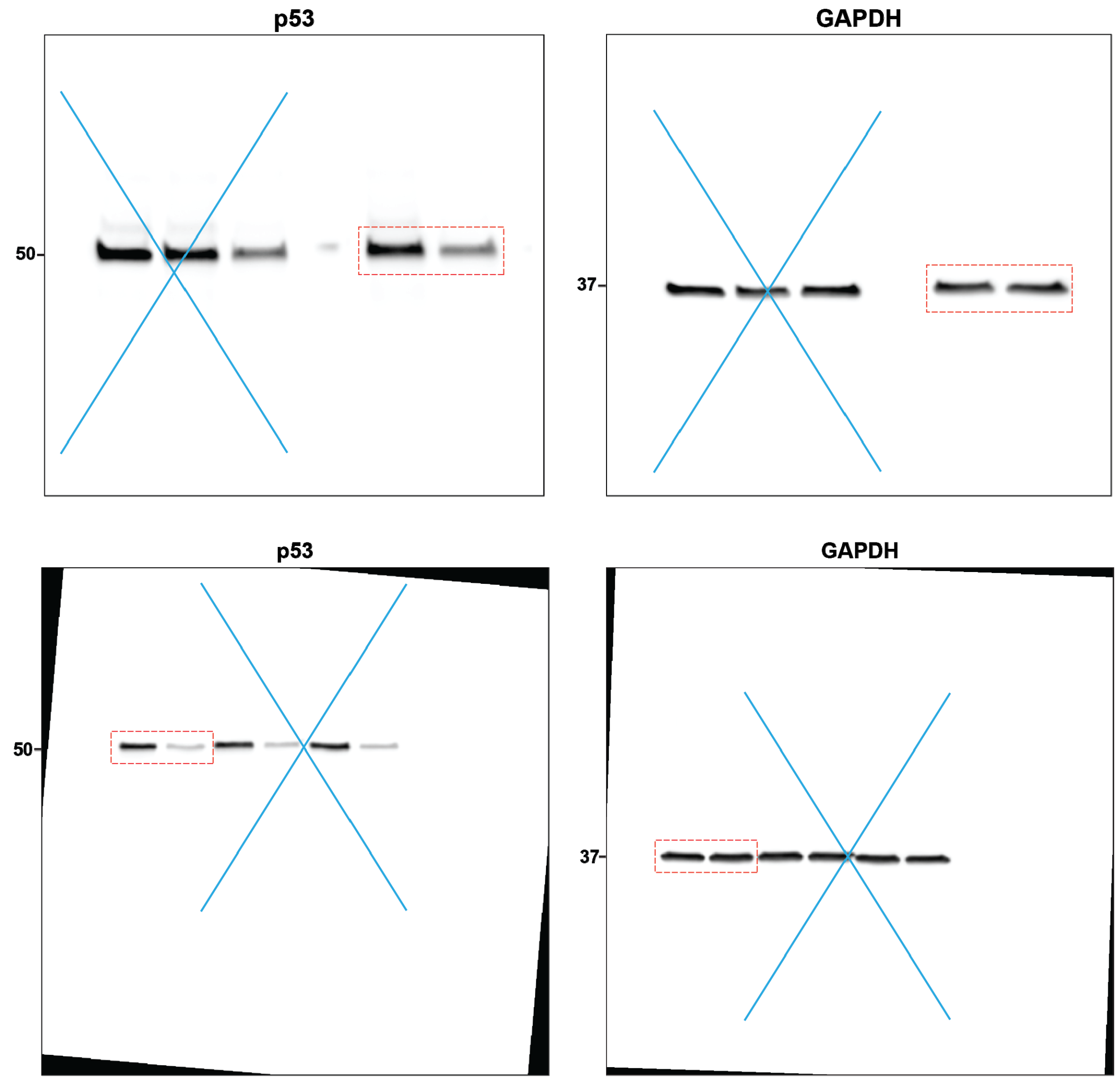

Supplementary Figure S18. Uncropped western blots used in Fig. S2m.
